# Study of the Segmental Dynamics and Ion Transport of Solid Polymer Electrolytes in the Semi-crystalline State

**DOI:** 10.3389/fchem.2020.592604

**Published:** 2021-01-13

**Authors:** Xi Chelsea Chen, Robert L. Sacci, Naresh C. Osti, Madhusudan Tyagi, Yangyang Wang, Jong K. Keum, Nancy J. Dudney

**Affiliations:** ^1^Chemical Sciences Division, Oak Ridge National Laboratory, Oak Ridge, TN, United States; ^2^Neutron Sciences Division, Oak Ridge National Laboratory, Oak Ridge, TN, United States; ^3^National Institute of Standards and Technology Center for Neutron Research, Gaithersburg, MD, United States; ^4^Department of Materials Science and Engineering, University of Maryland, College Park, MD, United States; ^5^Center for Nanophase Materials Sciences, Oak Ridge National Laboratory, Oak Ridge, TN, United States

**Keywords:** quasi-elastic neutron scattering (QENS), polymer electrolyte, ion transport, segmental dynamics, crystallinilty, thermal history, small angle X-ray scattering (SAXS)

## Abstract

Solid polymer electrolytes are promising in fulfilling the requirements for a stable lithium metal anode toward higher energy and power densities. In this work, we investigate the segmental dynamics, ionic conductivity, and crystallinity of a polymer electrolyte consisting of poly(ethylene oxide) (PEO) and lithium triflate salt, in the semi-crystalline state. Using quasi-elastic neutron scattering, the segmental dynamics of PEO chains confined between the crystalline lamellae is quantified, using Cole–Cole analysis. We show that the structural relaxation time, τ_0_, of PEO equilibrated near room temperature is six-fold longer than the same sample that had just cooled down to room temperature. This corresponds to a three-fold smaller ionic conductivity in the equilibrated condition. This work reveals that the segmental dynamics of semi-crystalline polymer electrolytes is very sensitive to thermal history. We demonstrate that quasi-elastic neutron scattering can be used to characterize the ion transport and segmental dynamics in the semi-crystalline state.

## Introduction

With growing demand of vehicle electrification, batteries with higher energy and power densities as well as improved safety features are in need. Solid polymer electrolytes with their low flammability, good thermal stability and excellent processability have great potential in enabling higher energy technologies such as lithium metal batteries.

The most commonly used polymer electrolytes is poly(ethylene oxide) (PEO) based electrolytes. In a model polymer electrolyte consisting of PEO and a lithium salt, lithium ions form coordination with the ether oxygen groups. Ion transport is assisted by the segmental motion of PEO through this coordination (Gray and Armand, 2011; Brooks et al., 2018). Therefore, quantification of the segmental dynamics of the PEO chains gives a direct prediction of the ion transport rate in the polymer electrolyte. In order to gauge the segmental dynamics of a polymer, a common method is to measure its glass transition temperatures (*T*_g_) through thermal and mechanical methods such as differential scanning calorimetry (DSC) and dynamic mechanical analysis (DMA). However, *T*_g_ measurements can only qualitatively compare different materials' segmental dynamics and cannot give quantified values of the segmental relaxation time. What's more, *T*_g_ measurements are often subject to sample geometry and measuring conditions. Quasi-elastic neutron scattering (QENS), on the other hand, is a powerful technique to directly measure the segmental dynamics of polymers, as the relaxation time of polymer chains can be quantified from QENS measurements. In polymer electrolytes, QENS has been used to resolve the relationship between segmental dynamics and ion conduction (Mao et al., 2000, 2001; Mos et al., 2000; Triolo et al., 2001; Fullerton-Shirey and Maranas, 2009; Sinha and Maranas, 2011; Sinha et al., 2012; Mongcopa et al., 2018; Chen et al., 2019). It is now established that the segmental mobility of PEO chains is slowed by the presence of lithium salts, due to salt-ether oxygen coordination (Mao et al., 2000; Fullerton-Shirey and Maranas, 2009; Mongcopa et al., 2018). Using QENS, different molecular motions can also be identified. In a polymer electrolyte containing PEO and a lithium bis(trifluoromethanesulfonyl)imide (LiTFSI) salt, at least two processes were identified: a slow process of a translational character and one or two fast processes of rotational characters (Mao et al., 2000).

One caveat of polymer electrolytes is that the host polymer PEO is a semi-crystalline material with a melting point of ~66°C (Hashmi and Chandra, 1995; Moreno et al., 2014). Since ion transport is aided by the segmental motion of the polymer chains, it takes place in the amorphous phase of PEO, as the segmental motion is largely frozen in the crystalline phase. Further, in the semi-crystalline state, the amorphous phase of PEO is confined between the crystalline lamellae, leading to low ionic conductivities (10^−5^ to 10^−8^ S/cm) at room temperature (Rao et al., 1994; Hallinan and Balsara, 2013). It is therefore important to understand the relationship between segmental dynamics, crystallinity, and ion conductivity of the polymer electrolyte in the semi-crystalline state.

Quantifying the segmental dynamics of polymer electrolytes in the semi-crystalline state can be challenging, as it is not only affected by the concentration and mobility of ions that are associated with the polymer chains, but also by the presence of the crystalline regions (Fullerton-Shirey and Maranas, 2009). The ion concentration also affects the degree of crystallinity and phase diagram of polymer electrolytes (Robitaille and Fauteux, 1986; Besner et al., 1992; Vallée et al., 1992; Lightfoot et al., 1993; Lascaud et al., 1994). Therefore, these parameters are not completely independent of each other.

In this work, we show that QENS can be used to quantify the segmental dynamics of PEO chains in the semi-crystalline state. We focus on one polymer electrolyte sample with a fixed salt concentration. The crystallinity of the polymer electrolyte is tuned by changing the thermal history of the sample. The segmental dynamics is quantified by calculating the structural relaxation time, τ_0_, of PEO chains using Cole–Cole function at specified temperatures. Through this experiment, the effect of crystallinity on the segmental dynamics of a polymer electrolyte is elucidated while the other parameters such as salt concentration and temperature are kept the same.

Our results indicate that τ_0_ of the polymer electrolyte equilibrated near room temperature for 1 week is six-fold longer than τ_0_ at the same temperature, but equilibrated only for hours. This resulted in a three-fold smaller ionic conductivity in the long-time equilibrated condition than the as-cooled condition. To our knowledge, the segmental dynamics of polymer electrolytes in the semi-crystalline state has not been quantified. This work shows that Cole–Cole function can be used to extrapolate segmental dynamics of polymers in the confined semi-crystalline state. Further, it demonstrates that the segmental dynamics may exhibit large variations depending on the thermal history of the sample. As the polymer electrolyte in a battery operates in cool-heat temperature cycles, the non-equilibrium nature must be taken into account.

## Experimental

### Preparation of Polymer and Composite Electrolytes

All materials were pre-dried in a vacuum furnace inside an Ar-filled glovebox for accurate weight measurements. PEO (Aldrich, average *M*_*w*_ = 600,000 g mol^−1^) and lithium trifluoromethanesulfonate (LiTf, Aldrich, 97%) were mixed in deionized water in a calculated ratio that resulted in molar ratio of [Li^+^]:[EO] = 1:16. The mixed polymer electrolyte solution was freeze-dried in a FreeZone 4.5 L benchtop freeze-dryer for 5 days and transferred into a glovebox antechamber where it was further dried for 16 h. We followed these rigorous drying steps to ensure complete removal of water from the sample (Peng et al., 2020). The complete removal of water was confirmed by infrared spectroscopy and thermal gravimetric analysis.

The freeze-dried polymer electrolyte was hot-pressed inside a glovebox at 100°C with light pressure applied for 1 h. The sample was pressed into a 200 μm thick template, which was calculated to be the optimum thickness for QENS measurements (~90% transmission to avoid multiple scattering events). The pressed film was annealed in a vacuum furnace inside the glovebox at 100°C for 4 h. The film was subsequently used for both QENS and ionic conductivity measurements.

### QENS Measurements

To prepare QENS samples, a piece of dried polymer electrolyte film with the dimension of 3 cm × 5 cm × 200 μm was cut and sealed in a flat aluminum can with indium wires in an Ar filled glovebox. Care was taken to make sure the entire area inside the can was filled with sample.

QENS measurements were conducted at the backscattering spectrometer (BASIS) (Mamontov and Herwig, 2011) beamline at the Spallation Neutron Source (SNS) at Oak Ridge National Laboratory. BASIS instrument was operated in a standard configuration [Si (111) analyzer crystals selecting the final wavelength of neutron = 6.267 Å] providing a fine energy resolution of 3.5 μeV, a dynamic range of ±120 μeV and a *Q* range of 0.3–1.9 Å^−1^. *Q* is the magnitude of the scattering vector defined as *Q* = 4π sin(θ/2)/λ, where θ is the scattering angle and λ is the wavelength of the incident neutrons. The sample was cooled to 20 K using Closed Cycle Refrigerator (CCR) to measure the instrument resolution function. QENS spectra were collected at 305, 335, and 365 K.

The same sample was also measured at the NG2 high-flux backscattering spectrometer (HFBS) (Meyer et al., 2003) beamline at NIST Center for Neutron Research (NCNR). QENS spectra were collected at 363 K and the instrument resolution function was collected at 20 K. Data were recorded over a dynamic range of ±15 μeV and a *Q* range of 0.25–1.75 Å^−1^.

Note that QENS signal predominantly comes from the hydrogen present in PEO. The measured scattering intensity is given by the following equation:

(1) $$\begin{array}{l} I\left(Q,E\right)=\left[p_{1}\left(Q\right)\delta \left(E\right)+\left(1-p_{1}\left(Q\right)\right)S\left(Q,E\right)\right]⊗R\left(Q,E\right)\\ \;+B\left(Q,E\right) \end{array}$$

In Equation (1), *p*_1_(*Q*) and δ(*E*) correspond to elastic incoherent scattering factor (EISF) and a Dirac delta function, respectively, and represent the elastic contribution to the measured spectra. 1−*p*_1_(*Q*) is the weight of QENS part contributing to the overall scattering intensity, *S*(*Q, E*), is the dynamic structure factor and represent dynamic processes within the sample. These two terms are convoluted with instrument resolution, *R*(*Q, E*). An additional linear background term, *B*(*Q, E*), is added to the model to take into account fast processes that are outside instrument dynamic range.

### Ionic Conductivity Measurements

The ionic conductivity of the polymer electrolyte was measured in an AA cell setup. A 1/2-inch diameter disk was punched out from the polymer electrolyte film and sandwiched between two pieces of copper foil followed by two stainless steel rods of the same diameter. The cell was sealed in two layers of heat shrink tubing. Impedance was measured in an AA battery holder by applying an alternating current of 6 mV amplitude within a frequency range of 1 MHz−50 mHz, using a Biologic SP-300. The sample was equilibrated at each temperature for 1.5 h before the impedance spectrum was collected. The resistance of the sample was obtained by fitting an equivalent circuit model and the ionic conductivity of the electrolyte was calculated accordingly.

### Differential Scanning Calorimetry (DSC) Measurements

DSC measurements were performed using a TA Instruments Q2000 DSC. A small piece of dried polymer electrolyte (~5 mg) was sealed in the DSC sample pan in an argon filled glovebox. Prior to DSC measurements, the sample was annealed inside the sealed pan at 100°C in the glovebox and equilibrated at room temperature for a week. DSC scans were run between −80 and 90°C at a 10°C/min scan rate under a nitrogen flow of 20 mL/min.

### Small Angle X-Ray Scattering (SAXS) Measurements

Small-angle X-ray scattering (SAXS) measurements were carried out on a Xenocs Xeuss 3.0 instrument equipped with D2+ MetalJet X-ray source (Ga Kα, λ = 1.341 Å). The samples (films) were aligned perpendicular to the direction of the X-ray beam (transmission mode) and the scattered beam was recorded on a Dectris Eiger 2R 4M hybrid photon counting detector with a pixel dimension of 75 × 75 μm^2^. The collected 2-dimensional (2D) SAXS images were circularly averaged and expressed as intensity vs. *q*, where *q* = (4πsinθ)/λ after subtraction of background scattering. The SAXS data were collected at two different sample-to-detector distances, i.e., 1.8 and 0.9 m and combined into one curve.

## Results and Discussion

**Figure 1:**
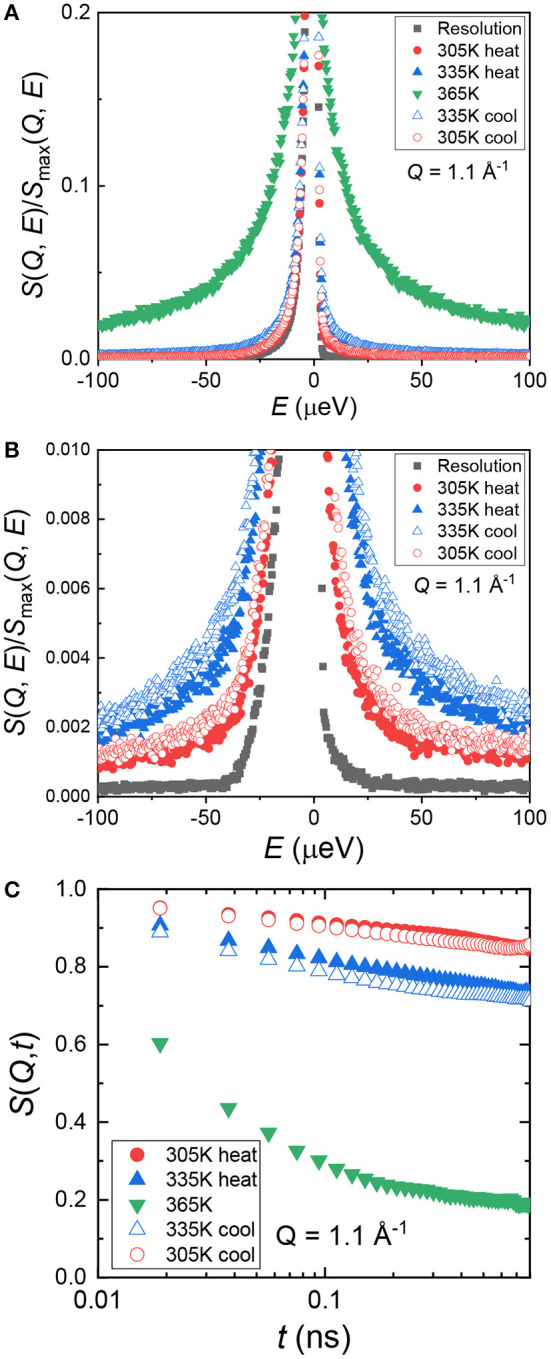
**(A)** Normalized dynamic structure factor, *S*(*Q, E*), as a function of energy, *E*, at a representative spatial scale (*Q* = 1.1 Å^−1^). **(B)** A zoomed-in view of panel **(A)** showing hysteresis of the heating and cooling cycle. (**C)** Intermediate scattering function, *S*(*Q, t*), as a function of time, *t*, at *Q* = 1.1 Å^−1^.

The model polymer electrolyte we chose to investigate was PEO and LiTf with an atomic ratio of [EO]:[Li^+^] = 16:1. We denote this sample as (PEO)_16_LiTf. The sample was annealed at 100°C and allowed to crystallize at room temperature in an argon glovebox for a week. During the QENS experiments, (PEO)_16_LiTf was first cooled down from room temperature to baseline temperature (20 K). Then, the sample was heated up from 20 to 365 K and cooled down to 305 K. Figures 1A,B show normalized dynamic structure factor *S*(*Q, E*) as a function of energy, *E*, at 4 temperatures, 20, 305, 335, and 365 K, at *Q* = 1.1 Å^−1^. The quasi-elastic line width increased with increasing temperature (Figure 1A), suggesting faster segmental mobility of PEO chains. Zooming in to the quasi-elastic region of two lower temperatures, 305 and 335 K, *S*(*Q, E*) exhibited hysteresis (Figure 1B). At the same temperatures, larger quasi-elastic broadening was observed in the cooling cycle than in the heating cycle.

We can re-express *S*(*Q, E*) in the time domain as the intermediate scattering function, *S*(*Q, t*). This was done by performing inverse Fourier transform followed by deconvolution from the resolution function through simple division in MantidPlot (Arnold et al., 2014). The result is shown in Figure 1C. In the time scale captured by BASIS instrument, 0.02–0.8 ns, (PEO)_16_LiTf only showed full relaxation behavior at 365 K. This temperature is well above the PEO melting temperature (~338 K). At 305 and 335 K, we did not observe complete chain relaxation as the amorphous region is confined between crystalline lamellae below the melting temperature of PEO. The segmental mobility of semi-crystalline PEO is significantly slower than the unconfined melt. This renders the standard Kohlrausch–Williams–Watts (KWW) function unsuitable for data fitting at 305 and 335 K.

To capture the dynamics of the PEO chains in this confinement, we used Cole–Cole function (Equation 2) to fit *S*(*Q, E*) in the energy space.

(2) $$\begin{array}{l} \;S\left(Q,\;E\right)\\ =\frac{1}{\pi E_{0}\left(Q\right)}\left[\frac{{\left(\frac{E}{E_{0}\left(Q\right)}\right)}^{-\alpha \left(Q\right)}\cos\frac{\pi \alpha \left(Q\right)}{2}}{12\;+\;{\left(\frac{E}{E_{0}\left(Q\right)}\right)}^{1-\alpha \left(Q\right)}\sin\frac{\pi \alpha \left(Q\right)}{2}+{\left(\frac{E}{E_{0}\left(Q\right)}\right)}^{2\left(-\alpha \left(Q\right)\right)}}\right] \end{array}$$

**Figure 2:**
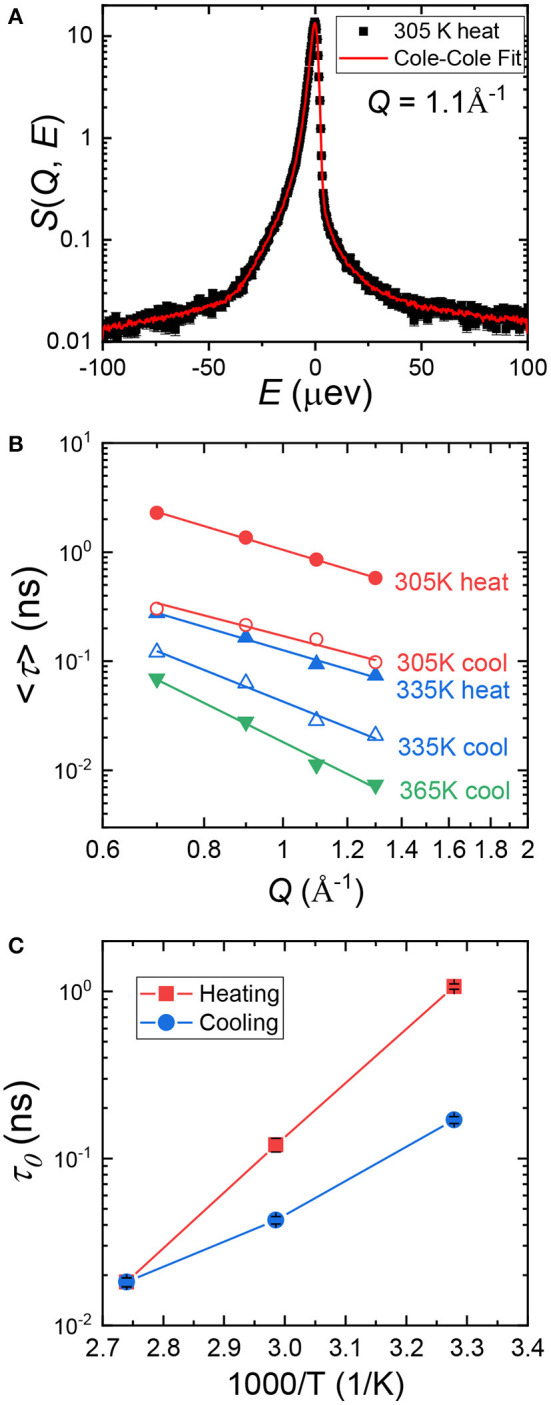
**(A)**, A representative Cole–Cole fitting (red curve) of *S*(*Q, E*) of (PEO)_16_LiTf at *Q* = 1.1 Å^−1^ at 305 K. **(B)** Average relaxation time, <τ>, as a function of *Q*. Data points were calculated from Cole–Cole fitting. The solid lines through the data points are fits using Equation (3). **(C)** Structural relaxation time, τ_0_, of (PEO)_16_LiTf as a function of inverse temperature, *T*, during heating and cooling cycles.

Cole–Cole distribution function has been successfully used to capture the dynamics in various confined systems (Cole and Cole, 1941; Gupta et al., 2016; Mamontov and O'Neill, 2017; Dyatkin et al., 2018). In Cole–Cole function, two parameters are fitted independently: *E*_0_(*Q*), the half width at half maximum of *S*(*Q, E*), and α(*Q*), the stretched exponential. When α = 0, Cole–Cole function becomes a Lorentzian function. We used Cole–Cole function to fit *S*(*Q, E*) at all three temperatures at each *Q* value to maintain data consistency. An example fitting curve is shown in Figure 2A. The average relaxation time, <τ>, can be calculated using the following relationship: $\bar{h}$/ <τ> = *E*_0_(*Q*), where $\bar{h}$ is the Planck Constant and is equal to 1.0541 × 0^−34^ J·s. In Figure 2B, we plot <τ> at each temperature as a function of *Q*. <τ> decreases with increasing *Q*, suggesting shorter relaxation time at smaller length scales. The *Q* dependence of <τ> can be obtained by fitting the data points with Equation (3), where τ_0_ is the structural relaxation time and γ is the power law exponent.

(3) $$\begin{array}{l} \langle \tau \rangle =\tau_{0}Q^{\gamma } \end{array}$$

Fittings using Equation (3) are shown by the solid lines in Figure 2B. Parameters extracted from the fits are shown in Table 1. At 365 K (melt state), γ = −3.69, very close to −4. When γ = −4, the polymer chain dynamics follows Rouse dynamics behavior for PEO chains (Genix et al., 2005; Tyagi et al., 2006; Brodeck et al., 2009). At 305 and 335 K, γ deviates from −4. As temperature decreases, γ deviates further. The lower values of γ indicate confinement of segmental dynamics within crystalline lamellae and the segmental dynamics do not follow Rouse behavior.

**Table 1:** Fitting results of *Q* dependence of <τ> using equation 2 (Cole-Cole function).

***T* (K)**	**γ**	***τ_0_* (ns)**
305 (heat)	−2.10 ±0.12	1.069 ± 0.038
335 (heat)	−2.51 ± 0.31	0.121 ± 0.012
365	−3.69 ± 0.29	0.018 ± 0.001
335 (cool)	−2.98 ± 0.17	0.043 ± 0.002
305 (cool)	−1.77 ± 0.23	0.170 ± 0.008

Comparing <τ> at 305 and 335 K during the heating and the cooling cycles, it is evident that <τ> is strongly affected by sample thermal history. <τ> is significantly smaller in the cooling cycle than in the heating cycle at the same *Q* values and temperatures (Figure 2B). Recall that the sample was allowed to crystallize in argon environment at room temperature for a week prior to the heating cycle measurement. Structural relaxation time, τ_0_, as a function of inverse temperature is shown in Figure 2C. At 305 K, τ_0_ in the heating cycle is 1.069 ns, six-fold larger than that in the cooling cycle, 0.17 ns. At 335 K, τ_0_ in the heating cycle is 0.121 ns, 3-fold larger than that in the cooling cycle, 0.043 ns. Both <τ> and τ_0_ data suggest higher segmental mobility of PEO chains in the cooling cycle, below the melting point.

**Figure 3:**
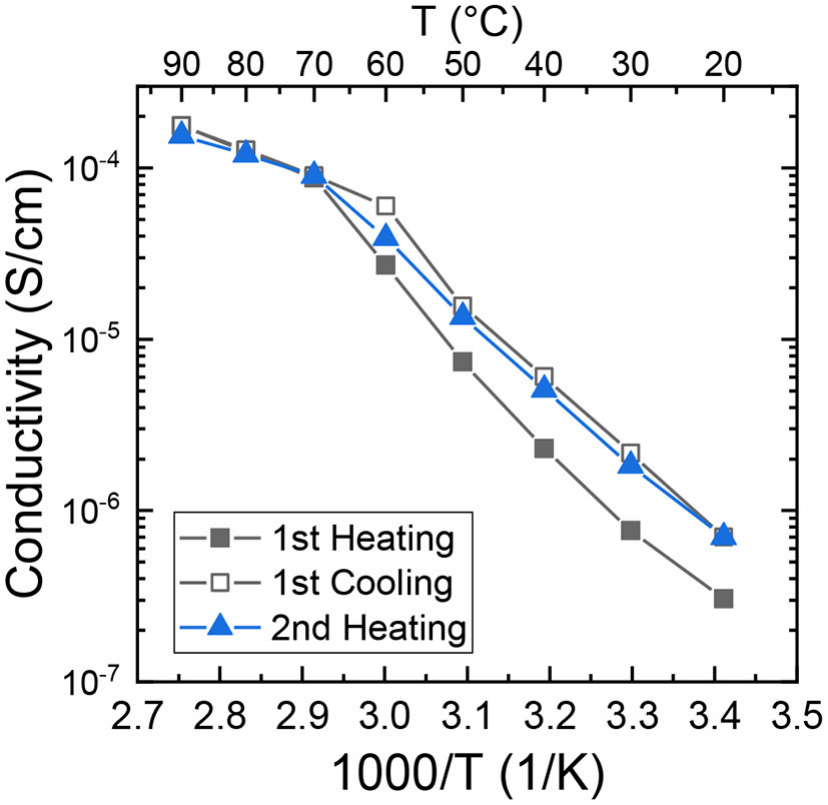
Ionic conductivity of (PEO)_16_LiTf as a function of inverse temperature.

We measured the ionic conductivity, σ, of (PEO)_16_LiTf as a function of temperature. The sample was first melted in between blocking electrodes at 100°C and annealed for 4 h. The sample was then cooled down to room temperature and equilibrated in the conductivity cell for 1 week. Conductivity measurements during heating and cooling scans were performed in a temperature-controlled chamber (Figure 3). The sample was equilibrated at each temperature for 1.5 h before the impedance spectrum was collected. This equilibrium time is typical for a temperature sweep as it ensures the sample reaches the set point temperature at each measurement (Pandian et al., 2018; Merrill et al., 2020; Palmer et al., 2020). In the melt state (*T* ≥ 70°C), no hysteresis in σ was observed during temperature cycling. Below the melting temperature of PEO (*T* ≤ 60°C) there is a large difference in σ during the first heating and first cooling scans. At 30°C (303 K), σ in first cooling cycle is 2.2 × 10^−6^ S/cm, three-fold larger than that in the first heating cycle (7.6 × 10^−7^ S/cm). At 60°C (333 K), σ in first cooling cycle is 6.0 × 10^−5^ S/cm, two-fold larger than that in the first heating cycle (2.7 × 10^−5^ S/cm). The conductivity data is consistent with the segmental relaxation times shown in Figure 2, where larger relaxation times corresponds to a smaller conductivity. Little difference is observed between the cooling cycle, and second heating, except for around the melting temperature.

**Figure 4:**
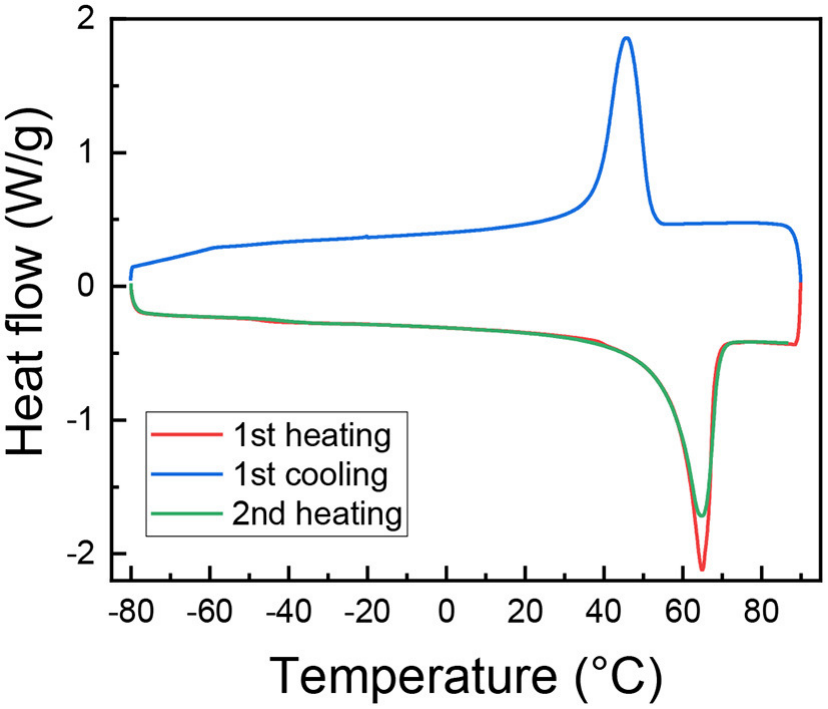
DSC thermogram of (PEO)_16_LiTf.

DSC scans were performed on (PEO)_16_LiTf, shown in Figure 4. Prior to DSC measurement, the sample was annealed in the DSC sample pan at 100°C and equilibrated at room temperature for a week in an argon glovebox. The endothermic peak around 65°C in the first and second heating scan represents the melting of crystalline PEO. The exothermic peak in the cooling scan represents the crystallization of PEO. The area under these peaks gives information of their degree of crystallinity, *X*_*c*_. It is observed that during the second heating, *X*_*c*_ decreased. The degree of crystallinity, *X*_*c*_, of PEO in (PEO)_16_LiTf calculated from the first heating scan was 54.9%, assuming the heat of fusion of 100% crystalline PEO was 203 J/g (Qiu et al., 2003). In the first cooling scan *X*_*c*_ = 50.8%, slightly lower than first heating. *X*_*c*_ calculated from the second heating thermogram was 52.5%. The higher ionic conductivity and segmental mobility observed in the first cooling cycle is the result of a decrease in *X*_*c*_, since the sample was not given enough time to fully crystallize. Note that the conductivity data are often reported during the first cooling or second heating cycles. This is mainly because in the first heating cycle the sample may not form good contact with the electrodes, if it wasn't previously annealed above the melting point of PEO on the electrodes. After annealing, it takes days for the samples to fully crystallize (Fullerton-Shirey and Maranas, 2009). Our results indicate that conductivity in the semi-crystalline state is strongly affected by the sample history.

We now have a discussion between the segmental dynamics, conductivity, and crystallinity of (PEO)_16_LiTf. Since 335 K is very close to the melting transition of PEO, we focus on data collected at 305 K, near room temperature. At 305 K, τ_0_ in the heating cycle is six-fold larger than that in the cooling cycle. Conductivity in the heating cycle is three-fold smaller than in the cooling cycle. A larger relaxation time corresponds to a smaller conductivity, and the changes in τ_0_ and conductivity are on the same order of magnitude. This qualitatively verifies the validity of using Cole–Cole analysis for the segmental relaxation of PEO chains under confinement.

**Figure 5:**
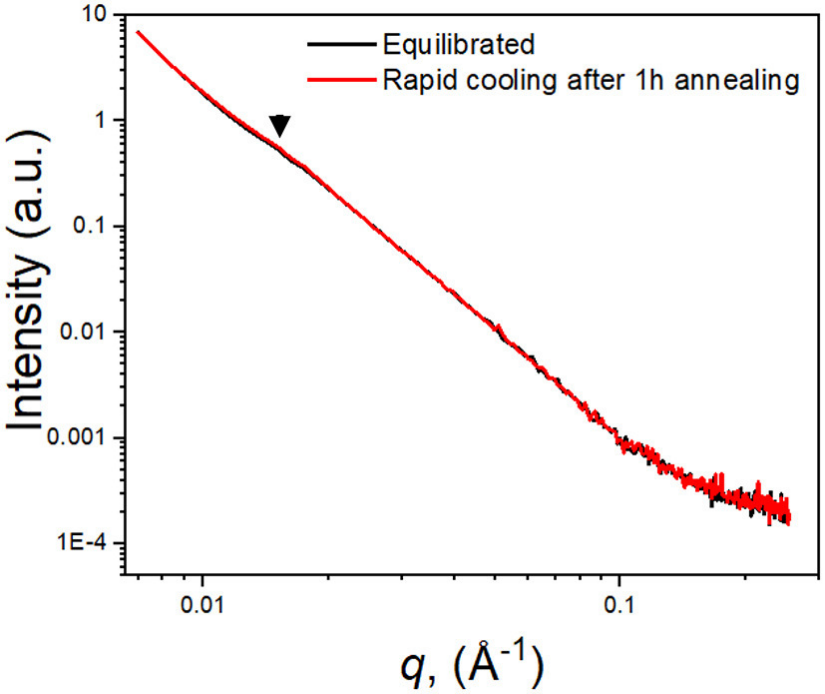
Small angle X-ray scattering of (PEO)_16_LiTf at room temperature. Black curve, the sample was allowed to equilibrate at room temperature for a week; red curve, the sample was annealed at 90°C for 1 h followed by rapid cooling to room temperature.

On the other hand, the difference between the degree of crystallinity is relatively small between the heating and cooling cycles, only 5%, compared to significant changes in both τ_0_ (300%) and conductivity (600%). We further investigated the crystallinity of (PEO)_16_LiTf by measuring the crystal lamellae distances using small angle X-ray scattering. Figure 5 compares the SAXS profiles of two (PEO)_16_LiTf samples with different thermal histories, both measured at room temperature. One sample (black profile) was annealed at 90°C and allowed to equilibrate at room temperature for a week. The other sample (red profile) was annealed at 90°C for 1 h and rapidly cooled to room temperature and the SAXS measurement was performed immediately. In spite of the different thermal histories, the two SAXS curves overlapped almost perfectly, where one peak was observed on both profiles, at q^*^ = 0.015 Å^−1^, indicated by black triangles. This peak was caused by the formation of PEO crystalline lamellae and their long-range ordering. The spacing, *L*_sp_ between the long-range ordered crystalline lamellae is calculated as *L*_sp_ = 2π/*q*. The calculated *L*_sp_ in both cases was 42 nm. The SAXS results suggest that thermal history difference did not result in a difference in the spacing of crystalline lamellae. We hypothesize that the difference between the morphology of the equilibrated sample and the rapid cooled sample may be the size, shape or distribution of the crystalline domains, which could not be captured by our SAXS instrument.

The discussion above highlights that it's challenging to capture the whole picture of the crystallization in a semi-crystalline polymer, with many parameters to consider, such as the degree of crystallinity, crystalline structure, grain size, distribution, etc. On the other hand, the segmental dynamics of the amorphous phase is very sensitive to the crystallinity of the sample, due to the connectivity between the crystalline phase and the amorphous phase and the confinement effects. This work highlights that QENS is a unique tool in that it not only gives information of the ion transport and segmental dynamics in the amorphous phase, it also indicates crystallinity changes in the crystalline phase.

## Conclusions

In this work, we investigated the segmental dynamics, ionic conductivity, and crystallinity of a polymer electrolyte consisting of PEO and LiTf, below its melting point. We demonstrate that QENS can be used to quantify the segmental dynamics of PEO chains in the semi-crystalline state, using Cole–Cole analysis, which, to our knowledge, has not been done. At 305 K, the structural relaxation time of (PEO)_16_LiTf that had been equilibrated for 1 week was six-fold longer than the same sample that had just cooled down to 305 K. Correspondingly, the ionic conductivity was three-fold smaller in the equilibrated condition. The crystallinity of (PEO)_16_LiTf did not show significant change given different thermal histories. We hypothesize that a change in the size, shape and distribution of the crystalline domains may have caused the significant change in the segmental dynamics and ionic conductivity in the amorphous phase. This work reveals that the segmental dynamics of semi-crystalline polymer electrolytes is very sensitive to thermal history. QENS not only can be used to characterize the ion transport and segmental dynamics in the amorphous phase, it can also be used to indicate changes in the crystalline phase, as the confinement effect significantly affects the segmental dynamics.

## Data Availability Statement

The original contributions presented in the study are included in the article/supplementary materials, further inquiries can be directed to the corresponding author/s.

## Author Contributions

XC: experimental design, sample preparation, and data collection—QENS, ion conductivity, DSC, data analysis, manuscript writing, manuscript editing. RS: experimental design, data collection—QENS, data analysis—QENS, manuscript editing. NO: experimental design, data collection—QENS, data analysis—QENS, manuscript editing. MT: experimental design, data collection—QENS, data analysis—QENS, manuscript editing. YW: data collection—DSC. JK: data collection—SAXS, data analysis—SAXS, manuscript editing. ND: experimental guidance, manuscript editing. All authors contributed to the article and approved the submitted version.

## Conflict of Interest

The authors declare that the research was conducted in the absence of any commercial or financial relationships that could be construed as a potential conflict of interest.
